# Mobile elements, zoonotic pathogens and commensal bacteria: conduits for the delivery of resistance genes into humans, production animals and soil microbiota

**DOI:** 10.3389/fmicb.2013.00086

**Published:** 2013-04-30

**Authors:** Steven P. Djordjevic, Harold W. Stokes, Piklu Roy Chowdhury

**Affiliations:** ^1^The ithree institute, University of TechnologySydney, NSW, Australia; ^2^New South Wales Department of Primary IndustriesCamden, NSW, Australia

**Keywords:** complex antibiotic resistance loci, plasmids, IS*26*, mercury resistance transposons, zoonosis, genomic islands, soil microcosm

## Abstract

Multiple antibiotic resistant pathogens represent a major clinical challenge in both human and veterinary context. It is now well-understood that the genes that encode resistance are context independent. That is, the same gene is commonly present in otherwise very disparate pathogens in both humans and production and companion animals, and among bacteria that proliferate in an agricultural context. This can be true even for pathogenic species or clonal types that are otherwise confined to a single host or ecological niche. It therefore follows that mechanisms of gene flow must exist to move genes from one part of the microbial biosphere to another. It is widely accepted that lateral (or horizontal) gene transfer (L(H)GT) drives this gene flow. LGT is relatively well-understood mechanistically but much of this knowledge is derived from a reductionist perspective. We believe that this is impeding our ability to deal with the medical ramifications of LGT. Resistance genes and the genetic scaffolds that mobilize them in multiply drug resistant bacteria of clinical significance are likely to have their origins in completely unrelated parts of the microbial biosphere. Resistance genes are increasingly polluting the microbial biosphere by contaminating environmental niches where previously they were not detected. More attention needs to be paid to the way that humans have, through the widespread application of antibiotics, selected for combinations of mobile elements that enhance the flow of resistance genes between remotely linked parts of the microbial biosphere. Attention also needs to be paid to those bacteria that link human and animal ecosystems. We argue that multiply antibiotic resistant commensal bacteria are especially important in this regard. More generally, the post genomics era offers the opportunity for understanding how resistance genes are mobilized from a one health perspective. In the long term, this holistic approach offers the best opportunity to better manage what is an enormous problem to humans both in terms of health and food security.

## COMMENSAL BACTERIA ARE A CONDUIT FOR ANTIBIOTIC RESISTANCE GENES ACQUIRED BY PATHOGENS

Human gastrointestinal flora is a vast, complex microcosm dominated by anaerobic Bacteroidetes and Firmicutes but peppered by less dominant species belonging to the Proteobacteria, Actinobacteria, and Verrucomicrobia (Arumugam et al., 2011). Many of these commensal organisms have not been cultured *in vitro*, yet they provide a myriad of metabolic pathways that impact on essential metabolic requirements for the host including food digestion, chemical detoxification, and nutrient uptake (Perez-Cobas et al., 2012). Importantly, they may also possess antibiotic resistance genes in great abundance (Marshall et al., 2009). Antibiotic treatment profoundly influences the balance of these organisms in the gut such that commensal Proteobacteria increase in abundance at the expense of Bacteroidetes and Firmicutes. This is potentially problematic because major clinical pathogens belong to the Proteobacteria. Functional metagenomic studies have shown that almost half of the antibiotic resistance genes derived from cultivable human gut isolates representative of the Proteobacteria display remarkable levels of nucleotide sequence identity (>90%) to resistance genes found in clinical pathogens. In contrast, antibiotic resistance genes cloned from culture-independent sampling techniques are evolutionarily distant from known resistance genes. These studies prompted speculation that dormant pathogens reside within populations of Proteobacteria in the gut and that unforeseen barriers may limit the spread of antibiotic resistance genes found in dominant gastrointestinal species (Firmicutes and Bacteroidetes) into cultivatable commensal and pathogenic species (Sommer et al., 2009). While the commensal flora in the gastrointestinal tract represent a significant source of antibiotic resistance genes and a conduit for the introduction of antibiotic resistant bacteria into the clinical environment, much remains to be learnt about how resistance genes track between these populations.

## THE HOSPITAL ENVIRONMENT

The hospital environment is only one small component of the microbial biosphere. The intermingling of bacteria of diverse genera and the mobile genetic elements they share occurs here as it does elsewhere. The hospital however is obviously important in the context of nosocomial infections since it represents the immediate patient environment. Nosocomial infections are a global problem and an increasing cause of death. In terms of the published literature, it has been shown that the hospital is a conduit for the spread of multidrug resistant pathogens. The most common cited sources of patient acquisition are by person to person contact (Markogiannakis et al., 2008) – either directly from another patient or via healthcare staff – or, by infection from medical equipment. In the second case, specialized equipment such as ventilators (Bouadma et al., 2012) and arthroscopes (Tosh et al., 2011) present particular challenges as they are commonly used on immuno-compromised patients or are difficult to clean. However, sometimes point sources of infection can be something as simple as contaminated “sterile” saline (Yu et al., 2000).

The general hospital environment – bed linen, curtains, sinks, and the like – is also a known repository of known multidrug resistant pathogens (Boyce, 2007; Otter et al., 2011; Marchaim et al., 2012). Implicit in the definition of a “nosocomial infection” is that the patient strain is identical to a strain recoverable from elsewhere in the hospital. This however fails to address what is perhaps a more fundamental question. Specifically, did the lateral gene transfer (LGT) events that gave rise to the specific suite of antibiotic resistance genes found in the pathogen in question occur outside the hospital environment with the same strain being subsequently imported into the hospital where it became established? Alternatively, did LGT and other events, which created a strain that was subsequently found to cause an infection, occur within the hospital context? The scenarios are not mutually exclusive and in reality both are likely to be important. Once established in a hospital, pathogenic strains can be very difficult to eradicate even with extensive, targeted costly cleaning and other efforts (Gastmeier and Vonberg, 2008). Cleaning can reduce the microbial burden transiently. However, if the primary sources of colonization, such as organic soil and residues in drains, are not assiduously removed, bacterial numbers can rebound very quickly even after treatment with the strongest disinfectants (Dancer, 2011). Organic residues also provide an additional protective environment, in that they can facilitate the formation of biofilms carrying pathogens identified as sources of nosocomial outbreaks (Hota et al., 2009).

The general hospital environments described above represent direct sources of infecting pathogens. In addition however, these same niches are likely to comprise complex ecosystems made up of some of the same diverse bacteria that are in food production, agricultural, soil, or aquatic environments beyond the clinical context. These bacteria will include, not just the strains that infect patients, but also the common commensal and environmental bacteria, described elsewhere in this review, that potentially act as conduits for the spread of complex antibiotic resistant gene loci (CRL). It therefore follows that the lateral transfer of CRL is just as likely to occur in microbial communities within the hospital as anywhere. This is especially likely given the intense localized use of antimicrobial agents in the general hospital environment.

It has been argued that the use of the disinfectants in the years leading up to the introduction of antibiotics may have been important in facilitating the introduction of gene mobilizing elements such as class 1 integrons into pathogens (Gillings et al., 2009). This was inferred as a consequence of consistently finding genes conferring resistance to various quaternary ammonium compounds (*qac*) being linked to class 1 integrons in diverse Proteobacteria, but not necessarily pathogens, in the absence of antibiotic resistance genes. Hospitals were major users of disinfectants in the years leading up to the antibiotic era. It could therefore be speculated that some of the key steps that initiated the formation of complex multidrug resistance loci began via gene exchange events involving commensal bacteria and pathogens, and took place in the general hospital environment. In a recent study, it was shown that the same plasmid-associated CRL recovered from nosocomial infection mediating bacteria were present in the same strains in the patient environment and that these strains were co-resident with non-pathogenic bacteria that carry the same loci (Betteridge et al., 2012).

## WHAT IS THE ROLE OF THE SOIL MICROCOSM IN ANTIBIOTIC RESISTANCE?

Antibiotic resistance genes are likely to have their origins in environmental bacteria that produce and release antibiotics as a means of influencing microbial populations with which they compete for nutrients. These observations are in line with the identification of wide spectrum of antibiotic resistance genes in 30,000-year-old Beringian permafrost sediments (D’Costa et al., 2006, 2011) and must have existed before the widespread anthropogenic application of antibiotics. Soil contains a vast microcosm and is home to the Actinomycetes that produce the majority of all naturally produced antibiotics. Non-pathogenic Proteobacteria derived from soil and aquatic environments carry class 1 integrons lacking antibiotic resistance gene cassettes and are close relatives of class 1 integrons found in clinically relevant Gram negative pathogens (Marshall et al., 2009; Vignaroli et al., 2012). Other important locations for antibiotic resistance genes include microorganisms in hospital wastewater, aquaculture and other aquatic environments, food animal manure ponds, and the gastrointestinal tract of mammals. These harbor vast numbers of bacteria and are important environments where genetic information is exchanged on mobile elements via LGT. Recently, evidence for the exchange of antibiotic resistance genes between environmental bacteria and clinical pathogens was obtained by culturing multidrug resistant Proteobacteria from soil environments and characterizing the CRL they harbor (Forsberg et al., 2012).

Lateral gene transfer represents a key mechanism by which naturally occurring antibiotic resistance genes captured by a mobile scaffold such as an integron, transposon, phage, plasmid, or chromosomal island move from an environmental source into clinically relevant bacterial species. Food animal production, aquaculture, clinical medicine, and various agricultural practices are the heaviest users of antibiotics. Significant quantities of administered antibiotics are excreted unchanged into the environment (Knapp et al., 2010; Heuer et al., 2011; Kristiansson et al., 2011; Marshall and Levy, 2011; Dantas and Sommer, 2012). These agents induce stress responses in the indigenous microbial communities promoting LGT (Baharoglu et al., 2010; Blazquez et al., 2012) that likely contribute to the sharing of resistance genes between microbial communities from divergent reservoirs (Forsberg et al., 2012). Large numbers of commensal bacterial populations carrying antibiotic resistance genes, which are derived from ruminant and other food animal production species, are shed into the environment. When this occurs, they have the opportunity to interact with microbial populations in the soil and in waterways and facilitate the exchange of genetic material.

## KEEPING UP WITH THE EVOLUTION OF CRL

It is generally believed that gene exchange mechanisms thwart approaches to trace the movement of CRL through multiple intermediate hosts (Marshall and Levy, 2011). While it is true that CRL may undergo change during transmission through different reservoirs and intermediate hosts, it is also true that CRL typically displays “signature sequences” that can be targeted by selecting suitable primer sets for polymerase chain reaction (PCR). For obvious clinical reasons there is considerable interest in being able to rapidly detect and characterize antibiotic resistance genes in pathogens. In the last 20 years or so, PCR has been a powerful method for targeting specific genes or gene families (Barken et al., 2007) but has probably had limited impact in helping stop the rise of antibiotic resistance in the clinical and non-clinical context. This is because the most widely used PCR strategies largely focus on the detection of, for example, class 1 integrons and the resistance gene cassettes they carry but ignore the genetic context where class 1 integrons reside (Levesque and Roy, 1993; Levesque et al., 1995). Since class 1 integrons are strongly associated with a multiple antibiotic resistance phenotype (Leverstein-van Hall et al., 2002), these PCR assays have been widely adopted. However, a detailed analysis of the genetic context surrounding the insertion sites of class 1 integrons has not enjoyed nearly as much attention. Many studies restrict the application of PCR-based assays to particular organisms or particular resistance genes or gene families. Such approaches are extremely useful in answering defined questions. These may include the extent to which specific resistance genes or elements have infiltrated into particular pathogens (Barguigua et al., 2013), or specific geographical regions or hospital populations (Shibata et al., 2003; Koratzanis et al., 2011) or for tracking the spread of a particular subtype of the multiple antibiotic resistant *Salmonella enterica* serovar Typhimurium DT104 (Threlfall, 2000). The use of PCR multiplexing strategies can even see this extended to diverse gene families (Grape et al., 2007; Mendes et al., 2007).

Unfortunately PCR assays such as those described above are not proactively helpful in preventing or limiting the spread of specific resistance genes. This is perhaps best exemplified most recently by the unchecked spread of NDM-1 (New Delhi metallo-beta-lactamase-1) globally. Since being first reported in 2009 (Yong et al., 2009) some 270+ publications (based on a PubMed search using “NDM-1” as a keyword) have appeared reporting some aspect of resistance strains carrying an NDM-1 gene. Most of these were detected or confirmed by the use of PCR and primers that target the gene. Approximately 21 of these publications are in the form of a stated “first report” in a particular region or organism. However, detection has not prevented spread since it would appear that the gene is happily becoming endemic to the region or organism concerned. There are several reasons why molecular gene detection on its own is not a strong preventative tool. Most obviously, targeted PCR requires knowledge of the existence of a gene to design primers to detect it. From a clinical perspective “knowledge of existence” usually means presence in a pathogenic clone that causes an actual human infection. Thus a question that could be asked is – if the existence, and likely abundance, of this gene and it properties had been known before it became a clinical problem, could the current crisis with respect to this gene have been better managed? Another problem is that gene detection on its own is not informative as to how genes are being spread when the genes in question are subject of high rates of lateral gene transfer. Thus, being informed about whether the first report of NDM-1 in Iran (Shahcheraghi et al., 2013) compared to the first report in South Africa (Lowman et al., 2011) was a consequence of the same or different mobilizing elements may be helpful in ongoing spread and surveillance management.

Whether the same or different, knowledge of the resistance gene/element combinations present beyond the clinical context could be additionally helpful in understanding how newly emerging genes (in a clinical context) make their way through the biosphere. To us, it is surprising that there is not a greater focus on more detailed examination of resistance gene context. Knowledge of genetic context will have far greater implications for developing strategies to better predict the emergence of multiply drug resistant (MDR) pathogens and identify their source. CRL and the plasmids that carry them are mosaic in structure and contain genes whose origins are derived from different environmental niche. There is no doubt that efforts to lower the prevalence of MDR bacteria in hospitals will require concerted efforts in veterinary and agricultural arenas and in aquaculture to lower the use of antibiotics.

New generation sequencing efforts should aim to complete the sequences of plasmids, transposons, and chromosomal islands that provide the scaffolds needed to house and mobilize CRL. While developments in new generation sequencing have made massive strides in assembling draft microbial genomes, the characterization of CRL and other mobile elements that carry reiterated sequences remains a challenge. As discussed elsewhere in this review, whole genome sequencing will likely make this easier in the near future. Despite the growing view that this will be a simple fix, there are a number of technological hurdles that must be overcome before we can assume this will be resolved using current high throughput sequencing approaches. Hints at the general efficacy of this approach are coming from whole sequencing of plasmids from relevant strains where it has been shown that certain plasmid backbones and acquired modular components with and without NDM-1 can be sourced from different regions (Poirel et al., 2011; Chen et al., 2012; Partridge and Iredell, 2012; Partridge et al., 2012).

Once established in bacteria important to humans, genetic elements acting in combination can spread resistance genes extraordinarily quickly. Since first found in 2009, NDM-1 has been mobilized to and between a remarkable variety of plasmids, transposons, and other elements (McGann et al., 2012; Partridge and Iredell, 2012). Analysis of this single important gene has also shown that strong selection can generate a multitude of rearrangements in a span as short as 3 years such that dissecting evolutionary pathways can be difficult (Toleman et al., 2012). The NDM-1 story also highlights the fact that humans can potentially greatly impact on the rate bacteria can evolve (Gillings and Stokes, 2012).

**FIGURE 1:**
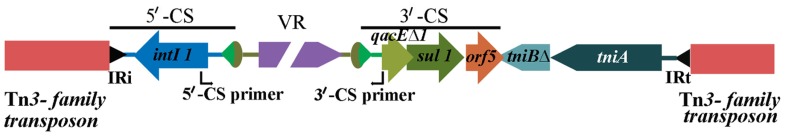
**Structure of a typical class 1 integron as seen in clinical isolates, embedded in a Tn*3*-family transposon**. The 5^′^-CS primer and 3^′^-CS primer are used to amplify across resistance gene cassette arrays.

Tracking genetic context through the microbial biosphere is as important for genetic elements as for individual genes. This is perhaps best exemplified by the class 1 integrons. Class 1 integrons are a special class of mobile genetic elements that include a site-specific recombination system and are important in the dissemination of antibiotic resistance genes in Gram negative bacteria (Hall, 2012). As a family of elements, integrons are quite diverse. However, it is the class 1 integrons (Hall, 2012) that have been most successful in passaging antibiotic resistance genes into pathogens. The recent evolution of class 1 integrons is quite complex (Gillings et al., 2008). Beyond the clinical context, class 1 integrons can be found in a variety of locations (Stokes et al., 2006) and in such cases the presence of cassettes that possess resistance genes is uncommon. In clinical isolates, detected class 1 integrons frequently possess and express multiple resistance gene cassettes, thus rendering isolates resistant to multiple antibiotics simultaneously. In addition, the same class 1 integrons also commonly have fixed conserved segments. These conserved segments are very often present regardless of the geographic or phylogenetic origin of the host pathogen. Also, these conserved segments flank the inserted cassette arrays such that a remarkably diverse suite of resistance genes and gene combinations can be located between them. These two conserved DNA sequences are known as the 5^′^-CS and 3^′^-CS (**Figure 1**; Stokes and Hall, 1989; Partridge et al., 2009).

With the above structure in mind, a PCR strategy was devised whereby primers designed to the respective flanking conserved regions allowed the recovery of resistance genes within the intervening variable region to be amplified (Levesque et al., 1995). The general strategy has become very broadly used in the past 18 years since the development of this PCR, as it allows resistance gene cassettes within integrons to be recovered in the absence of knowledge of the genes that may be present. While it is clearly an important epidemiological tool, the information that is derived is most useful if the limitations of the method are understood. Most clinical class 1 integrons are defective transposons and are not independently mobile. Nonetheless, they have become highly mobilized and are increasingly found embedded in other mobile elements such as the Tn*3* family of mercury resistance transposons. Consequently, class 1 integrons are just one component of CRL. Thus, a PCR (**Figure 1**) targeting a class 1 integron variable region can detect the presence of any (in theory) resistance gene cassette combinations at a specific location in particular strains, as long as the targets for the primers in the conserved segments are present. However, it can tell nothing about context beyond the integron and the mobile elements that contributed to its overall movement. Also, integrons can mobilize resistance gene cassettes by site-specific recombination. Consequently, it may be that different arrays, when compared, could be in the same genetic context. Equally, the same array recovered from different isolates may be in completely different contexts and thereby are derived via a different evolutionary pathway. In the case of *Pseudomonas aeruginosa*, lack of genetic context, despite hundreds of screens with this general method, has raised doubts as to whether resistance genes associated with integrons are predominantly located in plasmids or chromosomes (Stokes et al., 2012).

A second confounding factor in the use of conserved segment primers to study class 1-associated integron arrays is that the evolution of this mobile element is quite complex (Gillings et al., 2008). Specifically, the presence of the 3^′^-CS, the conserved segment located downstream of the genes in arrays, is not universally present. If absent, then any PCR reliant on this segment cannot generate a product. Thus, an array, if present, will go undetected. A cursory analysis of published studies where the authors have screened for class 1 integrons using standard primer sets show that they often report strains positive for the *intI1* gene but negative with primers that rely on the presence of the 3^′^-CS. Recent publications from our group support the contention that class 1 integrons with truncations in the 3^′^-CS are prevalent in clinical isolates of human and food animal origin (Dawes et al., 2010; Betteridge et al., 2011; Roy Chowdhury et al., 2011). Such integrons go undetected as PCR primers fail to generate amplicon/s because of the loss of a primer-binding site in the 3^′^-CS, although they harbor and express drug resistant genes. We refer to these as atypical class 1 integrons in contrast to the commonly described element shown in **Figure 1**, with both a 5^′^-CS and 3^′^-CS. The unintended consequence of assuming the presence of a 3^′^-CS is that the frequency of resistance arrays will be under-estimated. Despite the fact that the presence of a 3^′^-CS in particular is not universal, it nonetheless is common – at least in clinical isolates – leading to an outcome where diversity of structure and context is largely ignored. Given that class 1 integrons are common in soil Proteobacteria where they commonly lack a 3^′^-CS (Stokes et al., 2006) the tendency to assume that, when screening clinical isolates, the lack of a PCR product with a 3^′^-CS primer equates to a lack of an integron may be hampering our understanding of how this integron class moves resistance genes through the microbial biosphere.

How commonly does variable region PCR fail when a class 1 integron is present? This is a difficult question to answer since methodologies and choice of primers is inconsistent between studies. Where studies examine the genetic context of class 1 integrons a myriad of insertion sequence (IS) elements are found in close proximity. Some examples of IS elements found commonly in the genomes of members of Enterobacteriaceae include IS*1352*, IS*1326*, IS*6100,* IS*CRs,* IS*Ecp1*, and IS*26*. However, for the purposes of this review, we will focus only on examples where IS*26* has played a significant role in the evolution of CRLs. These elements are well-known to induce deletions adjoining the site of their insertion and play an important role in the formation of atypical class 1 integrons (Ploy et al., 2000; Dawes et al., 2010; Venturini et al., 2010). In one early study making use of targeted PCR methodology for a survey (Rosser and Young, 1999) about one-third of isolates lacked gene segments considered part of the 3^′^-CS. Other more recent studies report a similar frequency of atypical class 1 integrons (Kerrn et al., 2002; Guerra et al., 2003; Antunes et al., 2006; Hammerum et al., 2006; Dawes et al., 2010). However, in another study all class 1 integrons possessed a 3^′^-CS at least to the extent that a variable region was recoverable (Kim et al., 2011). Comparisons between different studies are nearly impossible since other variables including, targeted species and environment type confound meaningful analysis. Other studies have used the presence of aspects of the 3^′^-CS as the defining feature of a class 1 integron (Holzel et al., 2012) meaning that other class 1 integron types would go undetected. This is especially counter-productive to comparing human clinical isolates to non-human ones as class 1 integrons without a 3^′^-CS are likely to be more common in the latter (Gillings et al., 2008). In regards to assessing the resistance gene pool and diversity, class 1 integrons that lack a 3^′^-CS also carry resistance genes and knowledge of their abundance and distribution may contribute to better management of resistance gene spread (Dawes et al., 2010; Betteridge et al., 2011; Roy Chowdhury et al., 2011).

## IS PCR USEFUL FOR TRACKING CRL?

**FIGURE 2:**
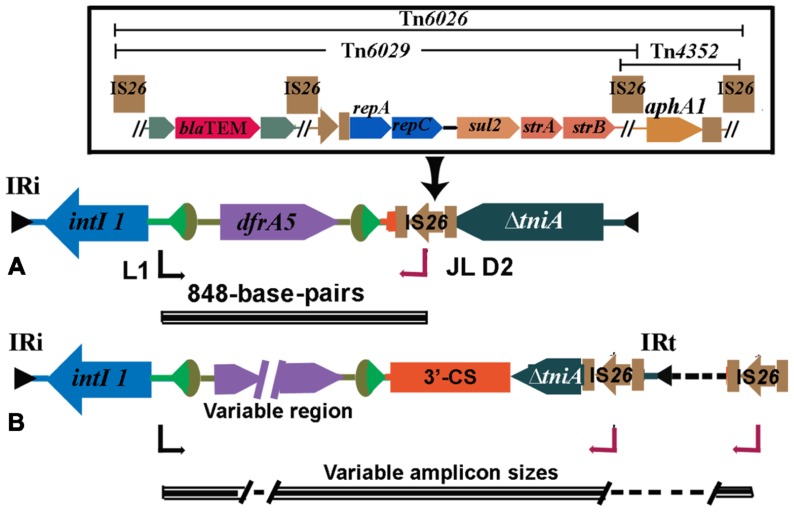
**(A)** Structure of atypical class 1 integrons found frequently in Australia. A characteristic 848 base-pair long PCR amplicon spanning *intI1*and IS*26* is generated when this structure occurs. The IS*26* can be part of composite transposons Tn*6026* or Tn*6029* (detailed in the inset). **(B)** Structures found in MDR strains overseas (see **Table 1**).

Polymerase chain reaction can be useful in tracking CRL across wide geographic environments and among different bacterial populations. Several important examples of this are discussed below. We designed a PCR, referred to here as the *intI1*-IS*26* PCR, with a forward primer (L1) located in the class 1 integrase gene, *intI1* and a reverse primer in IS*26* (JL-D2). The PCR was designed to screen strains that carried a class 1 integron but lacked the *sul1* gene (**Figure 2**). IS*26* was suspected of being responsible for its loss and is likely to be located in close proximity to the 3^′^-CS (Dawes et al., 2010). The PCR is of epidemiological value because IS*26* is increasingly found associated with CRL in multiply antibiotic resistant bacteria. When it was applied to a collection MDR *E. coli* that carried a class 1 integron but failed to produce positive results with a PCR to amplify the resistance gene cassettes, the *intI1*-IS*26* PCR generated an 848-bp fragment. This fragment comprised the 5^′^ end of *intI1*, a *dfrA5* resistance gene cassette that encodes resistance to trimethoprim, 24-bp of the 3^′^-CS and the 3^′^ end of the transposase gene in IS*26*. These data confirmed that these strains carried atypical class 1 integrons with truncated 3^′^-CS. Consistent with this hypothesis all strains positive with the *intI1*-IS*26* PCR were negative for the *sul1* gene. Strains positive for the *intI1*-IS*26* PCR also carried features consistent with the presence of Tn*21* indicating that the atypical class 1 integrons were associated with a CRL (Dawes et al., 2010). One isolate, strain O6877, was an enterohemorrhagic *E. coli* (EHEC) O26:H^-^ from an elderly patient with bloody diarrhea in Australia (Bettelheim et al., 2003; Dawes et al., 2010; Venturini et al., 2010). The 848-bp amplicon was generated from an 111-kb MDR IncI1 plasmid (pO26-CRL). Sequence analysis of pO26-CRL showed that it carries *bla*_TEM_, *aphAI*, *strAB*, *sul2*, and *dfrA5* genes encoding resistance to ampicillin, kanamycin/neomycin, streptomycin, sulfathiazole, and trimethoprim, respectively, and a suite of virulence factors that are known to play an important role in the colonization of the ruminant gastrointestinal tract (Venturini et al., 2010; Eckert et al., 2011). Reports of plasmids carrying combinations of virulence and antibiotic resistance genes are increasing in frequency and pose serious threats to human health (Kingsley et al., 2009; Venturini et al., 2010; Ahmed et al., 2012; Dolejska et al., 2013).

## MOBILE RESISTANCE GENES FLANKED BY IS*26*

IS*26* is now recognized to play an important role in mobilizing antibiotic resistance genes. While it is found flanking individual antibiotic resistance genes including *bla*_TEM-1_ (Bailey et al., 2011) and *aphA1* (Wrighton and Strike, 1987), it is increasingly found in association with CRL. Tn*6026*, Tn*6029*, Tn*4352* (**Figure 2**) are but three of a number of unusual transposons that are flanked by direct copies of IS*26*. Tn*6029* comprises resistance gene sequences of divergent origins including *bla*_TEM-1_ (encoding resistance to ampicillin) derived from Tn*2* (Partridge and Hall, 2005; Bailey et al., 2011) and *repA*–*repC*–*sul2*–*strA*–*strB* which has its origins in RSF1010 (Yau et al., 2010). In Tn*6026*, Tn*4352*, and Tn*6029* overlap, sharing a copy of IS*26*. A mechanism by which Tn*6029* evolved has been proposed (Cain et al., 2010). Interestingly, an intermediate step in the proposed path postulated a structure that was recently described in a MDR *S. enterica* serovar Typhimurium isolated in Italy (Cain and Hall, 2012; Lucarelli et al., 2012). Tn*6026*/Tn*6029*-like have been described in (i) enterohemorrhagic O26:H^-^ *E. coli* strain O6877 from Australia (Venturini et al., 2010), (ii) pRSB107, a plasmid encoding multiple antibiotic resistance and virulence genes isolated from hospital effluent in Germany (Szczepanowski et al., 2005), and (iii) in *S. enterica* serovar Typhi (Holt et al., 2007) and *S. enterica* serovar Typhimurium (Cain et al., 2010) and is likely to be an important contributor to the problem of MDR. In pASL01a, an *E. coli* plasmid contributing to the rapid rise in trimethoprim resistance in healthy nigerian students we identified a Tn*6029*-like transposon. Putative transposons flanked by IS*26* have a propensity to localize to regions in close proximity to class 1 integrons and many can be detected using our diagnostic PCR simply by amending the cycling conditions to allow for long range amplicon production (**Figure 2** and **Table 1**). IS*26* transposons are often found within the boundaries of class 1 integrons associated with mercury resistant transposons such as Tn*21*.

**Table 1:** List of sequences in GenBank recovered from a blastn analysis using the 848 bp *intI1*-IS*26* amplicon sequence as a query.

Accession number	Organism and source	Isolation country	Amplicon size (bp)	Amplicon content
HM999791.1	*E. coli* c19, human	Australia	848	*dfr*-Δ3′-CS::IS*26*
EU914100.1	*E. coli*, strain 59, human	Australia	848	*dfr*-Δ3′-CS::IS*26*
EU914098.1	*E coli*, strain D22, bovine	Australia	848	*dfr*-Δ3′-CS::IS*26*
GQ259888.1	*E.coli*, pO26-CRL	Australia	848	*dfr*-Δ3′-CS::IS*26*
HM999790.1	*E. coli*, c26, human	Australia	848	*dfr*- Δ3′-CS::IS*26*
GU562437.2	*S.enterica* pSRC125	Australia	11,973	*dfrA5-qacE*Δ*1-sul1*-Tni-module-mer-module of Tn*21*-*strB,A*-IS*26*
GQ150541.1	*S. enterica* pSRC26	Australia	4074	*dfrA5-sul1-qacE*Δ*1-orf5*-IS*6100*-IS*26*
JX424423.1	*K. pneumoniae* pKDO1	Checz Republic	5350	*dfrA14*-hypothetical gene-IS*6100*-EcoRII endonuclease- DNA methylase-IS*26*
AJ851089.1	pRSB107, uncultured bacterium	Germany	5429	*dfr*-mobC-IS*6100*-macrolide operon-IS*26*
HM999792.1	*E. coli*, c92	Australia	2082	*dfrA7-qacE*Δ*1-sul1*::IS*26*
JQ480155.1	*E. coli,* pASL01a	Africa	2082	*dfrA7-qacE*Δ*1-sul1*::IS*26*
CP003301.1	*E. coli* O104:H4 str. 2009EL-2071, genomic DNA	Georgia	2082	*dfrA7-qacE*Δ*1-sul1*::IS*26*
CP003289.1	*E. coli* O104:H4 str. 2011C-3493, chromosome	USA/Germany	2082	*dfrA7-qacE*Δ*1-sul1*::IS*26*
CP003297.1	*E. coli* O104:H4 str 2009EL-2050, chromosome	Georgia	2082	*dfrA7-qacE*Δ*1-sul1*::IS*26*
AM412236	*S. enterica* Paratyphi A, pAKU-1	Pakistan	2342	*dfrA7-sulI*-IS*26*
AJ628353.1	*Salmonella* Enteritidis, plasmid 6/9	Ireland	6593	*dfrA1-aadA1-*IS*26-strB, A-sulI-repC-repA-*IS*26*

## PCR FOR TRACKING *SALMONELLA* GENOMIC ISLAND 1

*Salmonella enterica* serovar Typhimurium DT104 with resistance to ampicillin, chloramphenicol/florphenicol, streptomycin/spectinomycin, sulphonamides, and tetracycline is a globally dispersed clone (Threlfall, 2000; Threlfall et al., 2000). The resistance genes *aadA2*, *sul1*, *floR*, *tetA*(G), and *blaP1* are housed within In104 (Levings et al., 2005), a 13 kb complex class 1 integron that resides with a 43 kb genomic island known as *Salmonella* genomic island 1 (SGI1; Boyd et al., 2001). The arrangement of resistance genes within In104 is unusual in that there are two *attI1* sites into which gene cassettes can be incorporated because of duplications of parts of the integron conserved segments that arose during its evolution. In MDR *S. enterica* serovar Typhimurium the *aadA2* gene cassette occupies one *attI1* site and *blaP1* the other. Using standard PCR primers designed to amplify class 1 integrons, this arrangement produced two amplicons of 1.0 and 1.2 kb and for many years these characteristic pair of PCR products was used to track MDR DT104 (Ridley and Threlfall, 1998; Sandvang et al., 1998). While this approach provided meaningful epidemiological observations, it failed to provide data that would address questions relevant to structural variants of In104 and mobility of SGI1. Furthermore, this PCR was unable to differentiate between different serovars of *S. enterica* that carried identical or related copies of SGI1 such as MDR *S. enterica* serovars Paratyphi B dT^+^, Kiambu, and Derby that are globally dispersed (Levings et al., 2005; Mulvey et al., 2006; Djordjevic et al., 2009). PCR primers that detected the boundaries of In104 within SGI1 and the location of SGI1 in the chromossome of *S. enterica* (Boyd et al., 2002) provide insight into the mobility of SGI1 within *S. enterica* serovars and in other MDR pathogens including *Proteus mirabilis* (Ahmed et al., 2007) and structural variants of In104 (Boyd et al., 2002; Levings et al., 2005; Mulvey et al., 2006) and the reservoirs where they reside (Levings et al., 2006).

## GENOME SEQUENCING: THE STATE OF PLAY

Genome sequencing is increasingly becoming the method of choice for large scale molecular phylogenetic, epidemiological, and metagenomic studies of bacterial populations. Current approaches are useful for performing large scale single nucleotide polymorphism (SNP) and indel analyses of multiple genomes but often fail to provide adequate coverage of CRL. Read length is insufficient to span insertion elements and other duplicated sequences (Loman et al., 2012) resulting in multiple contigs. Typically contigs do not span across CRL because of the presence of IS elements. While new generation paired end sequencing methodologies strive to improve read length, single molecule sequencing technologies that are potentially capable of excessively long read lengths remain expensive and prone to high error rates. This is exemplified by following the sequencing of O104:H4 outbreak strains. The O104:H4 outbreak commenced in May 2011 in Germany and spread to France and caused 4320 cases of bloody diarrhea, 850 cases of hemolytic uremic syndrome and 82 deaths (Mariani-Kurkdjian and Bingen, 2012) and is the most severe outbreak of its kind to date. Draft genome assemblies of outbreak strains appeared within days providing great insight into the hybrid nature of the genome (Mellmann et al., 2011; Rasko et al., 2011; Rohde et al., 2011). More refined genomic analyses identifying SNPs among the German and French outbreak strains appeared in 2012 (Grad et al., 2012) but the first closed genome sequences were not published until late in 2012, 18 months after the outbreak occurred (Ahmed et al., 2012; **Figure 2** and **Table 1**). These analyses provide new insight into the molecular evolution of O104:H4 outbreak strains and comparisons with genome sequences of O104:H4 isolates recovered from case studies in Georgia that occurred earlier in 2009. This study showed that multiple lineages of *stx*^+^ enteroaggregative *E. coli* (EAEC) are circulating worldwide and that EAEC progenitor strains may have acquired the *stx*_2_ prophage on more than one occasion (Ahmed et al., 2012). Furthermore, the study provides unparalleled insight into other important laterally acquired genetic elements such as prophages that characterize these unusual hybrid pathogenic strains. Until next generation sequencing read length improves substantially, detailed analysis of plasmid sequences and other laterally acquired CRL will require time consuming and expert manual annotation.

## ANTIBIOTIC USE IN AGRICULTURE AND VETERINARY MEDICINE

Many infectious agents that threaten human health are zoonotic (Heymann and Dixon, 2012). The O104:H4 outbreak strain was well-publicized for displaying resistance to an extended spectrum of β–lactams due to the presence *bla*_CTX-M-15_ gene. O104:H4 outbreak strains are also resistant to ampicillin (Ap), streptomycin (Sm), sulfamethoxazole (Su), tetracycline (Tc), and trimethoprim (Tp; Gault et al., 2011). It was not possible to definitively determine the location and context of genes responsible for encoding the Ap–Sm–Su–Tc–Tp resistance phenotype from incomplete genome sequences that emerged within days of the outbreak. The Ap–Sm–Su–Tc–Tp pattern is increasingly reported among MDR *E. coli* and *S. enterica* strains isolated from food-producing animals (**Table 1**). Where the genetic context of the location of these genes is known they are frequently associated with transposons such as Tn*6029* and Tn*6026* often in association with Tn*21* and other mercury resistant transposons on plasmids (Szczepanowski et al., 2005; Holt et al., 2007; Dawes et al., 2010; Venturini et al., 2010; Labar et al., 2012). IS*26* is a feature of the CRL found in these studies and others including SGI1 (Doublet et al., 2009) and is increasingly playing a central role in the evolution and mobilization of antibiotic resistance genes *en block* in enterobacterial populations.

Recently we described O26:H^-^ EHEC strain O6877, isolated from a patient with hemorrhagic colitis. Strain O6877 displays resistance to ampicillin, kanamycin, streptomycin, sulfathiozole, tetracycline, and trimethoprim (Bettelheim et al., 2003) and carries a virulence plasmid (pO26-CRL) that encodes resistance to all these antibiotics except tetracycline. Sequence analysis of pO26-CRL identified a 22,609 bp derivate Tn*21* mercury resistance transposon that had transposed into the *traC* gene rendering the plasmid non-conjugative. The derivate Tn*21* transposon is flanked by 5 bp direct repeats indicating it arrived there by a typical transposition event (Venturini et al., 2010). The antibiotic resistance genes were located within an In2-like integron containing a *dfrA5* resistance gene cassette that encodes resistance to trimethoprim, followed by only 24-bp of the 3^′^-CS which abuts Tn*6026*. We are now witnessing the emergence of plasmids and chromosomal loci that concomitantly house combinations of virulence and antibiotic resistance genes (Mulvey et al., 2006; Kingsley et al., 2009; Venturini et al., 2010). Many of these are likely to evolve in commensal bacterial populations that inhabit the gastrointestinal tracts of food-producing animals but are also in humans and are a cause for great concern.

Plasmids play a key role in harboring important virulence genes (Crossman et al., 2010; Venturini et al., 2010; Eckert et al., 2011). Large subpopulations of Shigatoxigenic *E. coli* (STEC) carry the enterohemolysin gene, *ehxA* and these are frequently excreted in the fecal contents of cattle and sheep (Fagan et al., 1999; Djordjevic et al., 2001, 2004; Ramachandran et al., 2001, 2003; Hornitzky et al., 2002, 2005; Brett et al., 2003a,b). The *ehxA* gene is a reliable marker for the presence of virulence plasmids that carry a broad array of genes encoding toxins, proteases, adhesins, immune avoidance molecules, and proteins involved in biofilm formation and protection against redox agents (Brunder et al., 1999; Venturini et al., 2010; Eckert et al., 2011). While many of these have been shown to be important virulence factors in cattle (Van Diemen et al., 2005; Eckert et al., 2011) recent *in vitro* studies show EhxA activates human macrophages and induces them to release the proinflammatory cytokine IL-1β (Zhang et al., 2012). We have shown that mercury resistance transposons carrying CRL transpose onto EHEC virulence plasmids (Venturini et al., 2010; Venturini and Djordjevic, unpublished results) and we believe that such mechanisms are playing an important role in the spread of CRL among important zoonotic pathogens. An appraisal of the literature shows that MDR EHEC isolates displaying resistance to ampicillin, streptomycin, sulfamethoxazole, trimethoprim, and tetracycline are frequently reported (Schroeder et al., 2002; Singh et al., 2005; Cain et al., 2010; Dawes et al., 2010; Li et al., 2011; Hiroi et al., 2012) but the molecular analyses of where the resistance genes are located are lacking. Nonetheless, these observations suggest that mercury resistance transposons play an important (Gastmeier and Vonberg, 2008) role in the assembly and transmission of CRL among EHEC and other Gram negative pathogens (Szczepanowski et al., 2005; Cain et al., 2010; Venturini et al., 2010; Labar et al., 2012). Plasmids and chromosomal islands that house CRL are mosaic structures that often comprise regions of DNA with divergent origins. A detailed molecular analysis of the mobile molecular scaffolds that both house and mobilize CRL is likely to have far reaching implications for predicting the emergence of new MDR and pathogenic strains.

Until the emergence of the O104:H4 outbreak strain and related strains, it was widely accepted that there were five different pathotypes belonging to the diarrheagenic *E. coli*: EAEC, EHEC, enterotoxigenic *E. coli* (ETEC), enteropathogenic *E. coli* (EPEC), enteroinvasive *E. coli* (EIEC; Kaper et al., 2004). EHEC and STEC more broadly form part of the commensal flora of ruminants and are increasingly gaining resistance to multiple antibiotics and are frequently reported to contaminate retail meats(Marshall and Levy, 2011; Ju et al., 2012; Sheikh et al., 2012; Kluytmans et al., 2013). Importantly, EHEC also colonize and invade plant cells found in fresh vegetables (Fegan and Gobius, 2012). The O104:H4 outbreak strain represents a shift in the paradigm because it represents an example where virulence factors traditionally confined to EAEC and EHEC have merged generating a hybrid. While the O104:H4 strain is genetically related to an EAEC isolated in the late 1990s from a patient in central Africa with HIV, the German outbreak strain is reported to have gained plasmids carrying genes encoding AAF/I fimbria and a CRL encoding the ESBL *bla*_CTX-M-15_ gene and genes encoding resistance to ampicillin, streptomycin, sulfamethoxazole, trimethoprim, and tetracycline but lost a plasmid encoding the heat stable enterotoxin, AstA. Importantly, the outbreak strain also acquired two prophages including one encoding *stx*_2a_. Nucleotide sequence analysis showed these prophages shared close identity with prophages found in O157 EHEC (Muniesa et al., 2012). Is the O104:H4 outbreak the product of rare, unlikely molecular events or do they point to a future where pathogens carrying combinations of virulence genes previously considered residing solely within an *E. coli* pathotype occur with greater frequency? LGT events can rapidly change a commensal to a pathogen. It has been proposed that the prototypical ETEC isolate H10407 need only gain genes encoding toxins that induce the loss of fluid from enterocytes, fimbriae for adhesion to intestinal epithelium and a type 1 secretion system, all of which are plasmid encoded, to become a pathogen (Crossman et al., 2010). Like antibiotic resistance genes, most known virulence genes are acquired laterally. Commensal bacterial populations naturally encode resistance to a wide array of antibiotic and are increasingly recognized as major reservoirs for resistance genes that find their way into clinically relevant pathogens (Marshall et al., 2009).

How multiple antibiotic resistance evolves and the pathways it follows through food animals, aquatic environments, and in humans via mobile elements remains poorly understood because the focus has been to identify the repertoire of resistance genes without including sequence coverage of the regions flanking CRL. If serious inroads are to be made toward understanding the problem of multiple antibiotic resistance and better methods to track their movement on mobile genetic elements are to be developed, much more needs to be done to generate complete (closed) sequences of MDR plasmids. The same can be said for genome sequencing. Valuable SNP and indel analyses can be undertaken using incomplete genome sequences but a comprehensive understanding of the role of laterally acquired DNA in genome evolution will be lost because genome sequences remain unfinished in public databases. These shortcomings will gradually be addressed as read length improves in next generation sequencing platforms.

## Conflict of Interest Statement

The authors declare that the research was conducted in the absence of any commercial or financial relationships that could be construed as a potential conflict of interest.
